# Does the Polydimethylsiloxane Urethral Injection (Macroplastique^®^) Improve Sexual Function in Women, in Fertile Age, Affected by Stress Urinary Incontinence?

**DOI:** 10.3390/medicina59030580

**Published:** 2023-03-15

**Authors:** Maurizio Serati, Andrea Braga, Chiara Scancarello, Andrea De Rosa, Matteo Frigerio, Yoav Baruch, Marco Torella, Stefano Salvatore, Alessandro Ferdinando Ruffolo

**Affiliations:** 1 Department of Obstetrics and Gynecology, Del Ponte Hospital, University of Insubria, 21100 Varese, Italy; 2 Department of Obstetrics and Gynecology, EOC-Beata Vergine Hospital, 6850 Mendrisio, Switzerland; 3 ASST Monza, Ospedale San Gerardo, 20900 Monza, Italy; 4 Urogynecology and Pelvic Floor Unit, Department of Obstetrics and Gynecology, Tel Aviv Medical Center, Faculty of Medicine, Tel Aviv University, Tel Aviv 6997801, Israel; 5 Department of Obstetrics and Gynecology, Second Faculty, 80100 Naples, Italy; 6 Obstetrics and Gynecology Unit, IRCCS San Raffaele Hospital, Vita-Salute University, 20132 Milan, Italy

**Keywords:** urethral bulking agents, bulking agents, polydimethylsiloxane, Macroplastique, sexual function, stress urinary incontinence

## Abstract

*Background and Objectives*: Stress urinary incontinence (SUI) negatively affects women’s quality of life, including sexual function. The aim of the current study was to evaluate the effect of polydimethylsiloxane (Macroplastique^®^) on sexual function in women of fertile age affected by SUI. *Materials and Methods:* Single-center prospective study. Sexually active women of fertile age with symptoms of pure SUI, which were urodynamically proven, were submitted to intraurethral Macroplastique^®^ injection. At 6-months follow-up, their sexual function was evaluated with Female Sexual Function Index (FSFI), while the SUI cure rate was objectively assessed through a negative stress test and subjectively by a Patient Global Impression of Improvement (PGI-I) score < 3. The difference of coital incontinence prevalence was assessed between the baseline and the 6-month follow-up. Peri- and postoperative complications of Macroplastique^®^ injection were recorded and classified according to the Clavien–Dindo system. *Results:* Twenty-one women fulfilled inclusion criteria and were submitted to Macroplastique^®^ procedure. The concerning sexual function, desire, satisfaction, and overall FSFI score significantly improved. Since other domains were less impaired at the baseline, we could not assess significant improvement for all of them. We observed a complete regression of coital incontinence (0/21, 0%) in comparison with the baseline (5/21, 23.8%; *p* = 0.04). The objective SUI cure rate was 76% (16/21), while the subjective SUI cure rate was 80.9% (17/21). One woman developed de novo overactive bladder, and two women developed postoperative voiding dysfunction (self-solved in 24 h). *Conclusions:* The Macroplastique^®^ urethral injection was demonstrated to be safe and effective in improving sexual function in sexually active women of fertile age affected by pure SUI, urodinamically proven at 6-months follow-up.

## 1. Introduction

Stress urinary incontinence (SUI) is the most frequent type of urinary incontinence (UI), with an overall prevalence of 48% and ranging between 29–75% depending on age [1]. Indeed, a steady increase in moderate and severe urinary incontinence (UI) has been reported throughout the adult lifespan, [2] with a distinct peak around the menopausal age (mainly around the fifth and the sixth decade) [3], while other studies did not assess an evident menopausal peak [4]. However, isolated SUI declines into old age as mixed incontinence becomes relatively more common. SUI has been proved to negatively affect women’s quality of life, including the sexual function of up to 68% of patients due to several factors [5]. Indeed, it has been reported that the urinary leakage can irritate the vulvovaginal region leading to dyspareunia. Furthermore, self-esteem, sexual desire, satisfaction, lubrication, and orgasm may be negatively involved, determining sexual dysfunction [6]. Fear about anorgasmia, coital incontinence, and a low frequency of coitus usually impairs sexual life in women affected by SUI [6]. Furthermore, effects of SUI on sexual function seem to not be limited only to affected women, with the quality of the intercourses that has been reported to be impaired also in partners [7,8].

Nowadays, several conservative or surgical treatments for SUI are available, and an improvement in incontinence symptoms has been associated with an improvement of sexual life [9,10] and of the overall quality of life [11]. Although improving SUI improves sexual well-being, sometimes complications related to anti-incontinence procedures may affect sexual function [12]. After their introduction in 1996, mid-urethral slings (MUSs) have been passed through numerous Level 1 randomized controlled trials [13] and are considered as the standard of treatment in women affected by SUI [12]. However, in the last decade, safety concerns related to MUS emerged. Indeed, several specific complications related to synthetic non-absorbable materials transvaginally implanted, such as the erosion of the vaginal mucosa with the exposition of the sling, the infection of the surgical site, and the incidence of persistent pelvic pain, have been reported [14]. When vaginal exposure of the non-absorbable synthetic material occurs in women submitted to an MUS procedure, it may lead to dyspareunia and, consequently, sexual impairment [15]. Moreover, vaginal MUS implantation itself may cause neurovascular tissue damages with sensory loss, pelvic pain, and dyspareunia [15,16,17]. In order to avoid complications related to MUS procedures, the injection of urethral bulking agents (UBAs) has returned to the forefront of SUI treatment [18]. Nowadays, UBAs are considered as an alternative option to MUS and as a first surgical choice in patients with comorbidities, high anesthetic risk, and women who prefer a less invasive approach [19]. Although MUS was demonstrated to be more effective than UBA in treating SUI, the advantages of the latter were the reduced rate of complications [20]. Sexual function outcomes after UBA procedures have been scarcely evaluated in the literature. In a previous prospective multicentric study, the polydimethylsiloxane silicone (Urolastic^®^ Urogyn BV, Nijmegen, The Netherlands) was demonstrated to improve sexual function similarly to MUS at 6 and 12 months of follow-up [21]. Similarly, Itkonen Freitas et al. assessed a similar improvement of both sexual function and quality of life both in women who underwent polyacrylamide hydrogel-PAHG (Bulkamid^®^, Contura International A/S, Soeborg, Denmark) urethral injection and in women who underwent tension-free vaginal tape (TVT) procedure [22].

Polydimethylsiloxane (Macroplastique^®^, Cogentix Medical, Orangeburg, New York, NY, USA) was the first available UBA, approved by the Food and Drug Administration in 2006. Macroplastique^®^ is a minimally invasive bulking agent that demonstrated an objective cure rate of 47% and a subjective cure rate of 49% for SUI at over 3 years of follow-up [23], and a lower morbidity when compared with MUS [15]. Moreover, Macroplastique^®^ was demonstrated to be effective for the treatment of complicated types of SUI, such as recurrent SUI [24]. There are no trials that compared different UBAs each other, neither for SUI nor for sexual function improvement. However, a recent systematic review by Hoe et al. [25] showed the Macroplastique^®^ and the Bulkamid^®^ to present the longest-term success rate for SUI in comparison with the other types of UBAs, even if Bulkamid^®^ procedures demonstrated a superior safety profile, considering that some cases of urethral erosion have been described for the Macroplastique^®^ injection.

The aim of the current study was to evaluate the effect of the polydimethylsiloxane (Macroplastique^®^) on sexual function in women of fertile age affected by SUI.

## 2. Materials and Methods

This is a single-center observational analytical prospective study carried out from January 2020 to April 2022 at the Urogynecological Unit of the Del Ponte Hospital of Varese, Italy. During the study period, 67 women were submitted to UBA procedures for SUI treatment. All consecutive sexually active women >18 years old of fertile age (with regular menstrual cycles) complaining of pure SUI with proven isolated urodynamic stress incontinence (USI) received intraurethral bulking agent (polydimethylsiloxane, Macroplastique^®^) procedure. Exclusion criteria for the submission to the anti-incontinence procedure were: >pelvic organ prolapse (POP) I stage, according to the pelvic organ prolapse quantification (POP-Q) system, voiding dysfunction or a post-void residual volume > 100 mL, concomitant overactive bladder (OAB) symptoms or urodynamic diagnosis of detrusor overactivity (DO), recurrent urinary tract infections (rUTIs), sexually inactive women, previous history of radical pelvic surgery, or pelvic radiation. Eligible patients were counselled for Macroplastique^®^ urethral injection and MUS procedure in accordance with the available data in literature; the choice of the surgical method was based on the patient’s preference.

Preoperative evaluation included medical history, physical examination, urinalysis, and a complete urodynamic testing. At baseline, sexual function was evaluated with the Female Sexual Function Index (FSFI), a questionnaire composed of 19 questions to investigate seven final items (desire, arousal, lubrification, satisfaction, orgasm, pain, and a total score). Each item of the FSFI is evaluated on a 0–6 scale where lower score indicates a worse condition [26]. Coital incontinence was defined as the “complaint of involuntary loss of urine with coitus,” and its prevalence was assessed by interview. Sexually active women were defined as women who had intercourse in the previous 4 weeks. Physical examination was performed in the lithotomy position with maximal Valsalva maneuver to assess POP presence, according to the International Continence Society (ICS) POP–Q system [27]. Urodynamic test included uroflowmetry, filling cystometry, pressure/flow study, and Valsalva leak-point pressure (VLPP) measurement, and it was performed according to the urodynamic practice guidelines of the ICS [28]. Women who fulfilled the inclusion criteria were submitted to the intraurethral injection of Macroplastique^®^ [29]. All women signed an informed consent form for intraurethral bulking agent procedure, allowing us to anonymously use data for research purposes. The “Macroplastique^®^ Implantation System” included the Macroplastique^®^ implantation device (MID) and two needles. The MID is a sterile, 25 F, single-use device with a fluid drainage channel and three fixed needle entry ports. A measuring scale for determining urethral length during the procedure is printed on MID. We inserted the MID tip into the urethra and advanced the tip into the bladder until fluid flowed from the fluid drainage channel. Then, we withdrew the MID 15 mm, and we injected 2.5 mL of Macroplastique^®^ in the 6 o’clock position, 1.25 mL in the 10 o’clock position, and 1.25 mL in the 2 o’clock position at 2–3 mm of depth into the urethral mucosa [29]. Only one experienced urogynaecological surgeon (MS) performed all the urethral injections of Macroplastique^®^ according to the original technique. All procedures were performed under general anesthesia. Patients were allowed to go home the same day when comfortable at voiding. If the patient was unable to pass urine spontaneously within 3–4 h of the procedure, “in-and-out catheterization” with a catheter was performed to relieve any symptoms of urinary retention. If PVRs of >100 mL were detected, the patient was followed up in the hospital until voiding.

At the 6-month follow-up visit, medical history, physical examination, stress test, subjective outcome evaluated by the Patient Global Impression of Improvement (PGI-I) [30], and sexual function through the FSFI score were assessed. The PGI-I is a 1-to-7 scale, validated for SUI, that evaluates the effect of the intervention on urinary symptoms, where 1 corresponds to “very much better” and 7 corresponds to “very much worse” [30]. A PGI-I value < 3 was considered as SUI subjective cure, and the objective success was defined as the absence of urinary leakage during a stress test performed with a full bladder obtained by spontaneous diuresis (>400 mL at ultrasonographic measure) [31] in lithotomy and upright position. The terminology used to describe urinary symptoms is in accordance with the standardization document of the International Urogynecological Association and the International Continence Society-IUGA/ICS [32].

Peri- and postoperative complications were collected. At follow-up, women were questioned about late post-operative complications. Complications were classified according to the Clavien–Dindo system [33].

Primary objective of the study was to evaluate the effect of the Macroplastique^®^ procedure on sexual function in women affected by isolated USI at 6-month follow-up.

The study was conducted according to the Declaration of Helsinki.

### Statistical Analysis

The Statistical Package for Social Science (SPSS) version 21.0 was used for data analysis. The Kolmogorov–Smirnov test was used to analyze the normal distribution of variables (>0.05). Pearson chi-square test and the exact Fisher test were adopted for categorical variables. For continuous variables, the Wilcoxon Rank Sum Test was used. The statistical analysis was conducted at 95% confidence level.

## 3. Results

During the study period, 21 women who fulfilled the inclusion criteria were submitted to the Macroplastique^®^ procedure. The baseline characteristics of the study population are reported in Table 1.

All 21 women (100%) were not lost at 6-months follow-up and could be included in the study analysis. Differences in sexual function domains, assessed through the FSFI score, between the baseline and the 6-month follow-up are reported in Table 2. Desire, satisfaction, and overall FSFI score significantly improved at 6-months follow-up. Since the other domains were less impaired at baseline, we could not assess a significant improvement for all of them.

Moreover, we observed a complete regression of coital incontinence at 6-months follow-up (0/21, 0%) in comparison with the baseline (5/21, 23.8%; *p* = 0.04). The objective SUI cure rate, defined as a negative stress test at 6-months follow-up, was reported in 76% of the study population (16/21). The subjective SUI cure rate, defined as a PGI-I < 3, was reported in 80.9% of women (17/21).

The Clavien–Dindo classification of the complications [33] showed only two women (2/21; 9.4%) complaining of post-operative voiding dysfunction. However, both presented a spontaneous regression in 24 h (Table 3).

Moreover, one patient complained of de novo OAB symptoms and was treated through an administration of antimuscarinics for 3 months with a total resolution of the symptoms.

## 4. Discussion

The results of our prospective study demonstrated the Macroplastique peri-urethral injection to be effective in improving sexual function (specifically sexual desire and satisfaction domains) in sexually active women of fertile age complaining of pure SUI at 6-months follow-up. The overall FSFI score improvement is surely the main outcome observed in our study. Concerning the efficacy of anti-incontinence procedures on sexuality, researchers suggest that managing urinary symptoms could significantly enhance the overall sexual functions of both patients and their partners. Indeed, women affected by SUI can experience an improved sexual life without such nervousness and anxiety following an anti-incontinence procedure, and several studies have already confirmed MUSs to improve sexual function because of SUI improvement [34]. Recently, the PAHG, as well as MUS (tension free vaginal tape-TVT) procedures, was demonstrated improving sexual function [22]. Similarly, Latul et al. observed an improvement in sexual function after the use of polydimethylsiloxane urolastic (PDMS-U), which was comparable to the improvement following MUS [21]. It has been reported that a fear of malodorous and urinary incontinence during coitus are associated with an alteration of image and self-esteem responsible for a low frequency of sexual activity among incontinent women [35], with a heavy impact on sexual desire. Indeed, it is not surprising that around half of women affected by urinary incontinence may avoid intercourse due to their symptoms [36,37]. In our population of sexually active women, the improvement of SUI symptoms led to an improvement of sexual desire and of the satisfaction of the intercourse. Moreover, the mean overall post-operative FSFI score of 29.2 resulted to be higher than the cut-off of 26.55 related to the definition of sexual dysfunction according to the FSFI score validation document [26], highlighting the great impact of the Macroplastique^®^ on the sexual function improvement.

Furthermore, the Macroplastique^®^ procedure improved coital incontinence in our study population. Two main mechanisms of coital incontinence have been described, either associated to the penetration or to the orgasm. While coital incontinence related to the orgasm has been associated with urgency urinary incontinence, coital incontinence associated with penetration has been related to SUI due to the probable intrinsic dysfunction of sphincter, more frequently reported in women with urodynamic stress incontinence [38,39]. A 2015 revision of sexual outcomes after SUI surgical treatment highlighted an improvement of sexual function in patients with SUI that also presented coitus urinary incontinence [40]. Our results confirm these observations showing a complete resolution of the coital incontinence symptom across all the symptomatic women.

Our results confirm the efficacy of the Macroplastique^®^ on SUI symptoms. Indeed, 76% of women were objectively cured (through a stress test), while 80.9% felt subjectively cured with a PGI-I < 3 at a 6-month follow-up. Even if the assessment of success widely varies among studies, our data seem to be in in line, or sometimes higher in comparison, with the previous literature that reported a short-term efficacy between the 48% and the 84% [23,25]. The high success rate reported in our study may be related to several factors. Indeed, all the procedures described in our study have been performed by the same expert surgeon (MS). Moreover, we reported about women with pure SUI without urgency symptoms. Our follow-up was a short-term follow-up at 6 months and, while MUS durability has been assessed and confirmed at a long-term follow-up, UBA procedures are associated with an important deterioration of efficacy over time [25]. All these conditions may have helped in reaching an objective cure rate, sometimes higher than the ones described in previous studies.

Concerning the choice of the study population, we decided to include only women of fertile age who were sexually active. Indeed, we did not include menopausal women in order to not include women affected by the genitourinary symptom of menopause (GSM); indeed, vulvovaginal atrophy related to GSM has been reported to have a high prevalence in post-menopausal women, estimated between 50%, and 80% [41]. Moreover, it has been clearly established that sexual function is a multifactorial condition, and its impairment should not only be related to vulvovaginal health. For this reason, we decided to include only sexually active women in order to reduce the risk of other conditions that are able to impair sexuality. In any case, considering the results of our study, we maintain that in young and sexually active women, the impact of anti-incontinence procedures on sexual function should be always considered. Multiple studies have described de novo dyspareunia as a contributing factor for decreased sexual global quality following MUS surgery [42,43]. Indeed, several factors, such as sling exposure and neurovascular tissue damage due to vaginal surgery, may cause sensory loss, pelvic pain, and dyspareunia. The Macroplastique^®^ bulking procedure demonstrated to be effective in improving sexual function in a group of young sexually active women became an important alternative when one of the goals of the surgical procedure was the preservation or the improving of sexual health.

The Macroplastique^®^ procedure was confirmed to be a safe procedure, with few and always mild complications, classified as Clavien–Dindo Grade I or II, reported. Indeed, two women presented a voiding dysfunction (defined as a PVR > 100 cc) that self-resolved in 24 h, while one woman presented post-operative de novo OAB symptoms that resolved after 3 months of antimuscarinics therapy.

This study presents some limitations. All of the Macroplastique^®^ procedures have been performed by the same highly trained gynecologist with a relevant expertise in UBAs injection procedures. The sample size is small due to the strict inclusion criteria (women of fertile age and sexually active) and limits the power of the study analysis. Moreover, the single cohort study, without the enrollment of a control group such as women treated by MUS, can be considered an intrinsic limitation of such a design. Furthermore, the short follow-up of 6 months limits the possibility of extending the study results at a longer time. However, this is one of the first studies evaluating sexual function after UBA procedures, and the first one evaluating the effect of Macroplastique^®^ on sexual function in women affected by SUI. Moreover, our study, with its strict inclusion and exclusion criteria, performed in a well-selected population composed by women of a fertile age (in order to eliminate confounding factors for sexual impairment, such as the genitourinary symptoms of menopause) who were sexually active (in order to reduce the risk of other causes of sexual dysfunction) and affected by isolated and urodynamically proved SUI. This may be helpful in increasing the reproducibility of our study with the knowledge and evidence in this field.

## 5. Conclusions

The Macroplastique urethral injection was demonstrated to be safe and effective in improving sexual function in a population of sexually active women of fertile age who were affected by pure SUI, which was urodynamically proven at a 6-month follow-up. Moreover, our data confirm the efficacy of the Macroplastique procedure for the treatment of SUI. Therefore, the Macroplastique procedure should be considered in sexually active women complaining of SUI. Longer-term studies with larger sample sizes are needed to confirm the durability of the result of our prospective study.

## Figures and Tables

**Table 1:** Baseline characteristics of the study population.

	*N* = 21
Age, years	41 (30–49)
BMI, kg/m^2^	25.3 (23–28)
Obese (BMI ≥ 30 kg/m^2^)	3 (14.2)
Previous vaginal deliveries	1 (1–2)
Operative delivery (vacuum/forceps)	4 (19)
FDTV (mL)	180 (50–430)
CC (mL)	480 (220–500)
PDetMax during filling phase (cmH_2_O)	8.4 (3–15)
Qmax (mL/s)	21 (7–77)
I-OpenP (cmH_2_O)	23.4 (9–66)
PDetMax during voiding (cmH_2_O)	31.5 (10–75)
PDetQMax (cmH_2_O)	24.4 (8–60)
VLPP (cmH_2_O)	64 (24–91)

Data are expressed as absolute number (%) or median (interquartile range) BMI = Body Mass Index. FDTV = First Desire to Void; CC = Cystometric Capacity; PDetMax = Maximum Detrusor Pressure; Qmax = Maximum Flow; I-OpenP = Intravescical Opening Pressure; PDetQMax = Detrusor Pressure at Maximum Flow; VLPP = Valsalva Leak Point Pressure.

**Table 2:** FSFI scores before and after bulking agents.

FSFI Domain	Baseline	After Surgery	*p* Value
Desire	2.4 (1.2–5.4)	3.6 (1.2–6)	0.02
Arousal	4.2 (0–5.4)	4.2 (0–6)	0.11
Lubrication	4.8 (0–6)	4.8 (0–6)	0.87
Orgasm	5.2 (0–6)	5.2 (1.2–6)	0.33
Satisfaction	3.2 (0.5.2)	5.2 (0–6)	0.008
Pain	4.8 (0–6)	4.8 (0–6)	0.72
Total Score	22.40 (16.60–24.80)	29.20 (24.60–34.50)	0.02

Data are expressed as median (interquartile range). The Kolmogorov–Smirnov test was used to analyze the normal distribution of variables (>0.05). Wilcoxon Rank Sum Test was adopted for intragroup comparison (<0.05).

**Table 3:** Clavien–Dindo classification of complications.

Complication	*N* = 21	Treatment
Grade I - Voiding dysfunction	2 (9.4)	Observation with spontaneous resolution in 24 h
Grade II - de novo OAB	1 (4.7)	Antimuscarinics

Data are expressed as absolute number (%). OAB: Overactive Bladder.

## Data Availability

Available from the correspondent author under reasonable request.
